# Analysis of Flexural Strength and Brittleness of a Polyjet 3D‐Printed Denture Base Polymer

**DOI:** 10.1002/cre2.70228

**Published:** 2025-09-21

**Authors:** Gregory W. Bennett, Alex Kohler

**Affiliations:** ^1^ Department of Adult Restorative Dentistry University of Nebraska Medical Center College of Dentistry Lincoln Nebraska USA; ^2^ Student Nebraska Wesleyan University Lincoln Nebraska USA

**Keywords:** denture base, flexural strength, material jetting, printing

## Abstract

**Objectives:**

This study sought to understand the effects of print orientation on a novel 3D‐printing technology recently made available for the fabrication of dentures.

**Material and Methods:**

A total of 90 experimental samples (30 each at 0, 45, and 90 degrees) were printed using denture resin on a polyjet printer. The milled control samples (*n* = 10) were milled on a 5‐axis dental laboratory mill from a denture base disc. 96 samples were tested for flexural strength and extension at break using a 3‐point bend test on a universal testing machine. The data were analyzed using statistical software and evaluated for statistical significance using general linear models with a Dunnett test with α = 0.05.

**Results:**

The results showed that the flexural strength and extension at break were both affected by print orientation with the 90‐degree orientation being the lowest performing group for both properties. The 0‐degree and 45‐degree orientations both exceeded the ISO minimum flexural strength of 65 MPa, with mean flexural strengths of 88.18 and 73.53 MPa. The 90‐degree group mean was well below the standard at 28.12 MPa. The milled sample group mean was 65.18 MPa. Extension at break showed similar results with less variation in the printed groups. Mean extension at break of the milled samples group was 15.05 mm, the 0‐degree was 5.99 mm, 45‐degree was 4.72 mm, and 90‐degree was 1.92 mm.

**Conclusion:**

Flexural strength and extension at break were influenced by print orientation. A 0‐degree, or horizontal, print orientation yielded the highest values for flexural strength. The milled samples had significantly higher values for extension at break, but similar values for flexural strength.

## 1 Introduction

Prosthodontics is the dental specialty concerned with the restoration and replacement of missing or damaged teeth and oral structures (Layton 2023). Common prosthetic treatments include dentures, implants, crowns, and bridges, all aimed at restoring oral function and aesthetics (Alqutaibi et al. 2023). Among these, dentures remain the most widely used solution for partially or completely edentulous patients (Abdelnabi and Swelem 2024). Although the prevalence of edentulism has declined in industrialized nations due to improved oral health care and preventive strategies, a significant global need for oral rehabilitation persists, particularly among elderly populations (Carlsson and Omar 2010).

Traditional denture fabrication involves multiple clinical visits, contributing to increased chair time and treatment costs (Abdelnabi and Swelem 2024; Majeed et al. 2024). These limitations have driven the adoption of digital workflows that offer enhanced efficiency, accuracy, and patient satisfaction (Abdelnabi and Swelem 2024; Majeed et al. 2024). Computer‐aided design and manufacturing (CAD‐CAM) technologies enable clinicians to scan, design, and fabricate dentures with reduced clinical visits and faster turnaround times (Abad‐Coronel et al. 2023; Clark Brinton 2024). The two most common CAD‐CAM methods are subtractive milling and additive 3D printing (Lawson et al. 2024). Milled dentures, produced from pre‐polymerized PMMA blocks, offer high accuracy and fracture resistance and are widely used in clinical practice (Abad‐Coronel et al. 2023; Lawson et al. 2024; Graf et al. 2024; Saaedi et al. 2023). In contrast, 3D printing provides advantages such as cost‐effectiveness, design flexibility, and faster production, although its clinical use remains limited (Clark Brinton 2024; Lawson et al. 2024).

Various 3D printing methods construct objects layer by layer, and mechanical properties can vary significantly depending on material, technology, and print parameters (Abad‐Coronel et al. 2023; Graf et al. 2024). While 3D‐printed dentures show promise, clinical adoption is slowed by variability in material performance and concerns about long‐term durability (Clark Brinton 2024; Graf et al. 2024). Polymethyl methacrylate (PMMA) remains the standard for denture bases due to its favorable aesthetics, biocompatibility, and ease of processing (Altarazi et al. 2022; Gad and Abualsaud 2019). but it lacks ideal mechanical properties—most notably in its brittleness under functional stresses (Alqutaibi et al. 2023; Majeed et al. 2024). Recent studies have explored the incorporation of fibers, nanofillers, and hybrid additives to improve 3D‐printed PMMA, but enhancing one property often compromises another (Majeed et al. 2024; Altarazi et al. 2022; Gad and Abualsaud 2019).

One underexplored variable affecting the strength of 3D‐printed dental materials is build orientation—the angle at which objects are printed on the build platform (Mueller 2015). Orientation can influence material bonding, layer cohesion, and ultimately, mechanical strength. However, previous studies have shown conflicting results on the relationship between print orientation and flexural strength, suggesting that the effect may be material‐ or printer‐specific (Lawson et al. 2024; Mueller 2015; Al‐Dulaijan et al. 2023; Gad and Fouda 2023).

In this study, a newly released PolyJet 3D printer was used to fabricate denture base samples at 0°, 45°, and 90° orientations using a proprietary PMMA‐based resin. The mechanical properties of these samples were compared to those of milled controls. Specifically, we evaluated flexural strength and extension at break, a measure of material brittleness. An ideal denture base should combine high flexural strength with sufficient flexibility to avoid catastrophic failure during function or accidental drops (Alqutaibi et al. 2023; Sinha and Lawson 2024). The null hypothesis is that print orientation does not significantly affect the flexural strength or extension at break of PolyJet‐printed samples when compared to milled PMMA controls (α = 0.05). This study aims to contribute to a better understanding of the clinical viability of PolyJet 3D printing in denture base fabrication.

## 2 Methods

### 2.1 Sample Fabrication

**Figure 1:**
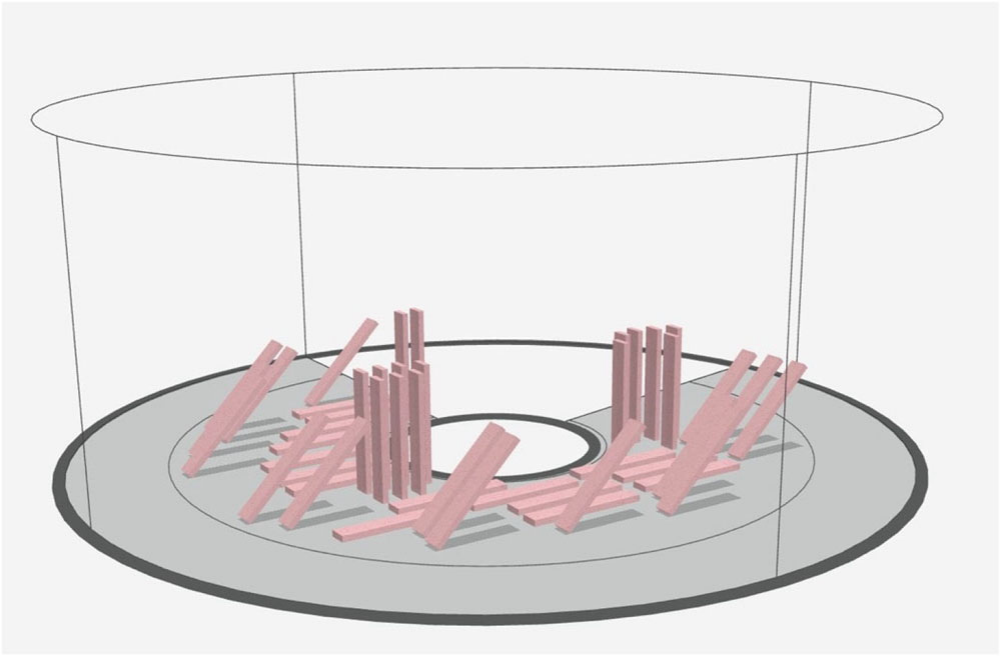
Flexural strength bar design file. Designed in accordance with ISO guidelines.

Rectangular bar‐shaped specimens were designed using 3D CAD software (Meshmixer, Autodesk) with dimensions of 65 mm × 10 mm × 3 mm, in accordance with ISO 20795‐1 specifications for denture base materials (Frei 2021). Using a PolyJet 3D printer (J5 DentaJet, Stratasys) and proprietary denture base resin (TrueDent, Stratasys), 90 samples were printed and divided into three groups based on build orientation (0°, 45°, and 90°; 30 samples per group). Post‐processing was performed according to the manufacturer's recommendations (Figure 1).

**Figure 2:**
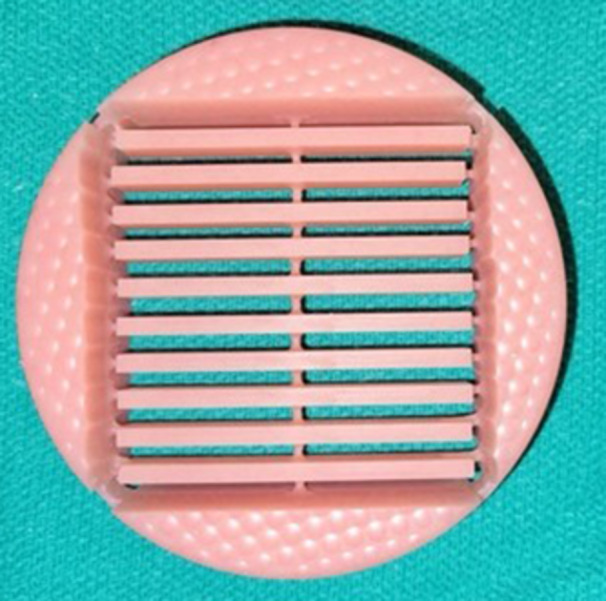
A milled denture base disc.

Control specimens (*n* = 10) were fabricated by milling from pre‐polymerized PMMA discs (Ivobase, Ivoclar Vivadent) using a 5‐axis dental laboratory mill (PM7, Ivoclar Vivadent) (Figure 2). All specimens were stored in distilled water at room temperature until testing to prevent dehydration.

### 2.2 Mechanical Testing

Before testing, each specimen was removed from water, thoroughly dried, and labeled. Specimen width and thickness were measured at the midpoint using a digital caliper. Mechanical testing was conducted using a universal testing machine (Instron 5500 R, Instron) configured for a three‐point bending test. Each specimen was placed on two supports spaced 50 mm apart and loaded at its midpoint until fracture occurred. This setup, known as a three‐point flexural test, is a clinically relevant method for assessing the flexural strength of denture base materials (Sinha and Lawson 2024).

**Figure 3:**
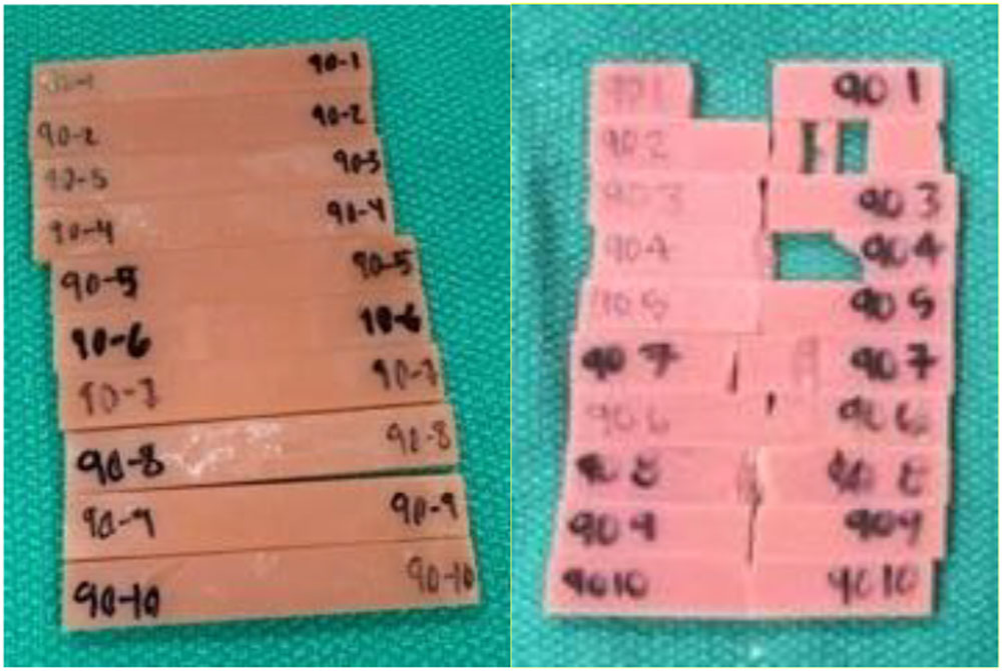
Photos of flexural strength bars before (left) and after (right) testing.

Fractured specimens were collected and stored for later disposal (Figure 3).

### 2.3 Data Collection and Calculations

Flexural strength (S) was calculated using the following formula:

$$S=\frac{3\mathrm{PL}}{2bd^{2}}$$

where

- *S* is the flexural strength (MPa).
- *P* is the maximum load at fracture (N).
- *L* is the support span (50 mm).
- *b* is the specimen width (mm).
- *d* is the specimen thickness (mm).

According to ISO 20795‐1, denture base materials must achieve a minimum flexural strength of 65 MPa. In addition to flexural strength, extension at break (in mm) was recorded for each specimen as a measure of brittleness.

### 2.4 Statistical Analysis

All statistical analyses were performed using SPSS software (version 29, IBM). Descriptive statistics including means, standard deviations, and range (minimum and maximum) were calculated for each group. A generalized linear model (GLM) was used to compare flexural strength and extension at break between experimental groups and the milled control. Post‐hoc comparisons were conducted using Dunnett's test to assess differences between each printed orientation and the control group. A significance threshold of α = 0.05 was applied for all tests.

## 3 Results

A total of 96 samples were tested, 86 printed and 10 milled. Two samples each from the 45‐degree and 90‐degree groups were non‐usable due to printing defects that resulted in bars of inappropriate dimensions (Table 1).

**Table 1:** Descriptive statistics.

	Flexural strength				Extension at break			
	Mean	Maximum	Minimum	Standard deviation	Mean	Maximum	Minimum	Standard deviation
Milled	65.18	77.23	53.88	7.05	15.05	19.39	9.94	3.30
0 Degree	88.18	105.69	36.75	18.37	5.99	9.82	1.85	1.96
45 Degree	73.53	85.43	43.03	8.88	4.72	5.66	2.52	.66
90 Degree	28.12	41.33	17.29	5.57	1.92	2.78	1.16	.39

### 3.1 Flexural Strength

Flexural strength varied across the orientations (Figure 3). The lowest mean flexural strength was in the 90‐degree orientation (28.12 MPa). The highest mean flexural strength for a printed group was the 0‐degree orientation (88.18 MPa). The milled group had a mean flexural strength of 65.18 MPa. The general linear model showed that there was a statistically significant difference between groups (Table 2). Post‐hoc Dunnett‐*t* tests (Table 3) showed significant differences for 2 of the comparisons. The 90‐degree, or vertical, orientation was significantly lower than the milled (*p* < 0.001) The mean flexural strength of the 0‐degree orientation was significantly higher than for the milled (*p* < 0.001). The mean flexural strength of the 45‐degree orientation was not significantly different from the milled control (*p* = 0.126).

**Table 2:** General linear model of flexural strength showing significant differences between groups.

Tests of between‐subjects effects – Flexural strength					
Source	Type III sum of squares	*df*	Mean square	*F*	Sig.
Corrected model	56140.771^a^	3	18713.590	130.465	< 0.001
Intercept	317617.031	1	317617.031	2214.322	< 0.001
Orientation	56140.771	3	18713.590	130.465	< 0.001
Error	13196.261	92	143.438		
Total	462511.434	96			
Corrected total	69337.033	95			

^a^ R squared = 0.810 (adjusted R squared = 0.803).

**Table 3:** Dunnett test showing significant differences between 0‐degree and 90‐degree from the milled control.

Multiple comparisons						
Dunnett *t* (2‐sided)^a^						
Orientation	Orientation	Mean difference	Std. error	Sig.	95% Confidence interval	
					Lower bound	Upper bound
0 Degree	Milled	22.9992*	4.37322	< 0.001	12.8630	33.1353
45 Degree	Milled	8.3511	4.41209	0.126	−1.8752	18.5773
90 Degree	Milled	−37.0589*	4.41209	< 0.001	−47.2852	−26.8327

*Note:* Based on observed means. The error term is mean square (error) = 143.438.

* Dunnett *t*‐tests treat one group as a control, and compare all other groups against it.

^a^ The mean difference is significant at the 0.05 level.

### 3.2 Extension at Break

**Figure 4:**
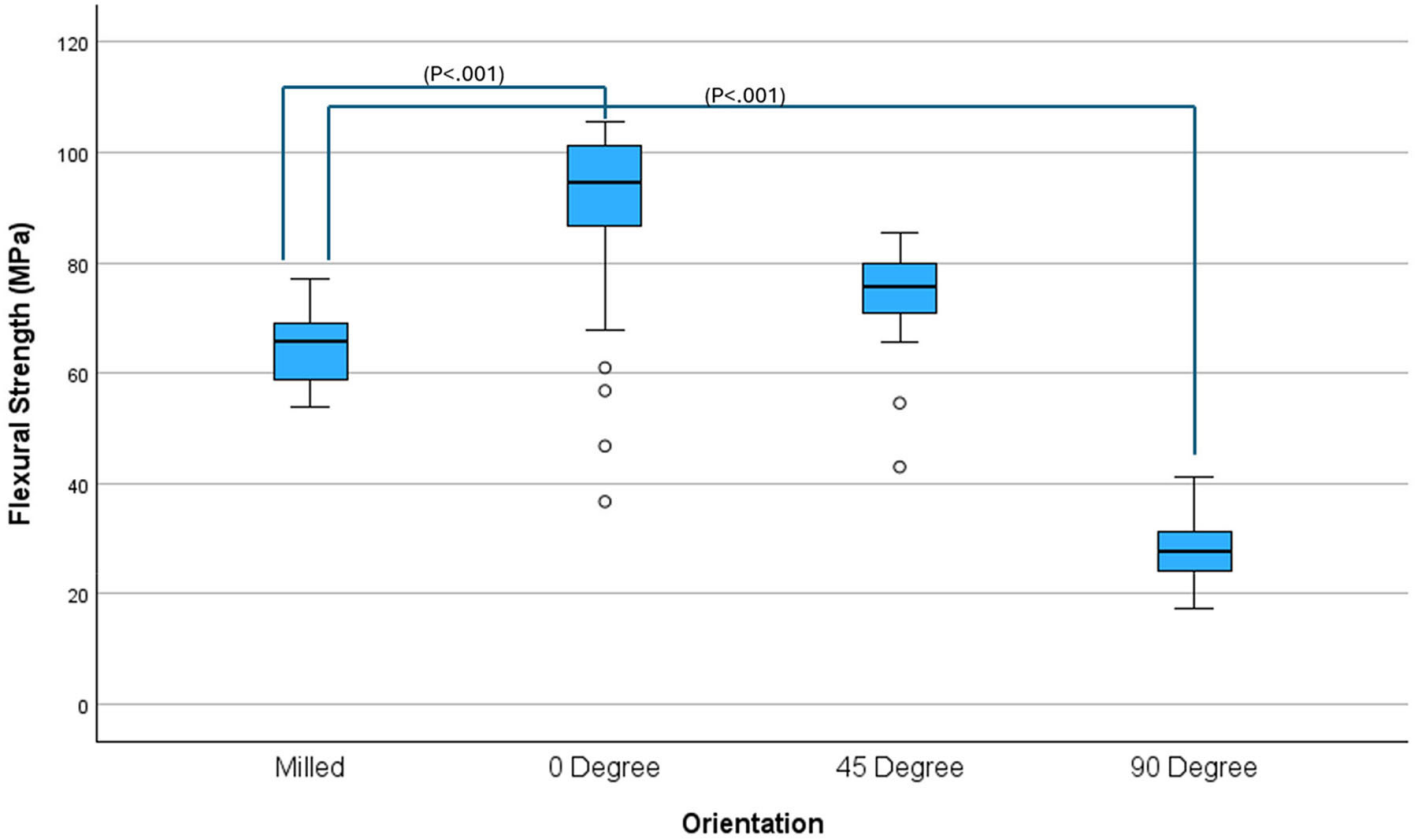
Flexural strength.

Extension at break showed less variation than flexural strength (Figure 4). The lowest mean extension at break was in the 90‐degree group (1.92 mm) and the highest mean extension at break was in the milled group (15.05 mm). The general linear model showed that there was a statistically significant difference between groups (Table 4). Post‐hoc Dunnett‐*t* tests (Table 5) showed significant differences between all printed groups compared to the milled group (*p* < 0.001).

**Table 4:** General linear model of extension at break showing significant differences between groups.

Tests of between‐subjects effects extension at break					
Source	Type III sum of squares	*df*	Mean square	*F*	Sig.
Corrected model	1294.888^a^	3	431.629	176.688	< 0.001
Intercept	3742.191	1	3742.191	1531.871	< 0.001
Orientation	1294.888	3	431.629	176.688	< 0.001
Error	224.746	92	2.443		
Total	4293.854	96			
Corrected total	1519.634	95			

^a^ R squared = 0.852 (adjusted R Squared = 0.847).

**Table 5:** Dunnett test showing significant differences between 0‐degree and 90‐degree from the milled control.

Multiple comparisons extension at break						
Dunnett t (2‐sided)^a^						
(I) Orientation	(J) Orientation	Mean difference (I‐J)	Std. error	Sig.	95% confidence interval	
					Lower bound	Upper bound
0 Degree	Milled	−9.0680*	.57072	< 0.001	−10.3908	−7.7452
45 Degree	Milled	−10.3318*	.57579	< 0.001	−11.6664	−8.9973
90 Degree	Milled	−13.1376*	.57579	< 0.001	−14.4721	−11.8030

*Note:* Based on observed means. The error term is mean square (Error) = 2.443.

* The mean difference is significant at the 0.05 level.

^a^ Dunnett *t*‐tests treat one group as a control, and compare all other groups against it.

## 4 Discussion

The present study aimed to determine the effect of build orientation on the mechanical properties of denture base samples fabricated using a newly released PolyJet 3D printing system. This printer uses a material jetting technique, in which photopolymer droplets are deposited and cured with UV light to form a 3D object layer by layer (Abad‐Coronel et al. 2023). The material used TrueDent Resin is a proprietary, FDA‐cleared (Class II) polymer designed for dental applications, including dentures, crowns, and bridges, and is reported to have an ultimate flexural strength of ≥ 85 MPa (Stratasys 2023). By comparison, the milled control material used in this study has a manufacturer‐reported flexural strength of 78 MPa (Frei 2021).

ISO 20795‐1:2013 recommends testing fracture toughness only when impact resistance is claimed by the manufacturer (Standardization IOf 2013), this property was not evaluated here, as TrueDent Resin does not advertise impact resistance. This aligns with a broader trend in the literature, which reveals a relative scarcity of data on the fracture toughness of 3D‐printed denture base polymers (Coldea et al. 2025). As the dental field continues its shift toward digital fabrication, including additive manufacturing, understanding these mechanical properties is increasingly critical to ensuring the clinical viability of emerging materials and workflows (Clark Brinton 2024).

Prior studies examining the effects of print orientation on denture base strength have produced conflicting results, often depending on the specific printing technology and resin used. For instance, Altarazi et al. found that SLA‐printed samples oriented at 90° had the highest flexural strength, (Altarazi et al. 2022) while Al‐Dulaijan et al. reported that 0° orientations yielded superior strength in DLP‐printed specimens (Al‐Dulaijan et al. 2023). Interestingly, in both studies—and in the current one—the 45° orientation consistently produced intermediate flexural strength values. Zeidan et al. compared milled, heat‐polymerized, and 3D‐printed denture bases (fabricated at a 45° angle), and reported that milled resins outperformed printed ones across all metrics (Zeidan et al. 2023).

In this study, the 0° print orientation outperformed all other printed groups and the milled control in terms of flexural strength, though it also demonstrated the greatest variability. This variability may be attributed to the distinctive mechanics of the PolyJet printer, which employs a rotating build platform governed by a polar coordinate system, rather than the more common XYZ Cartesian configuration. In traditional DLP systems, entire layers are exposed simultaneously, whereas the PolyJet process may introduce inconsistencies in droplet placement depending on the sample's orientation relative to the direction of rotation. These differences highlight the need for further investigation into the interaction between printer mechanics and print orientation (Figure 4).

**Figure 5:**
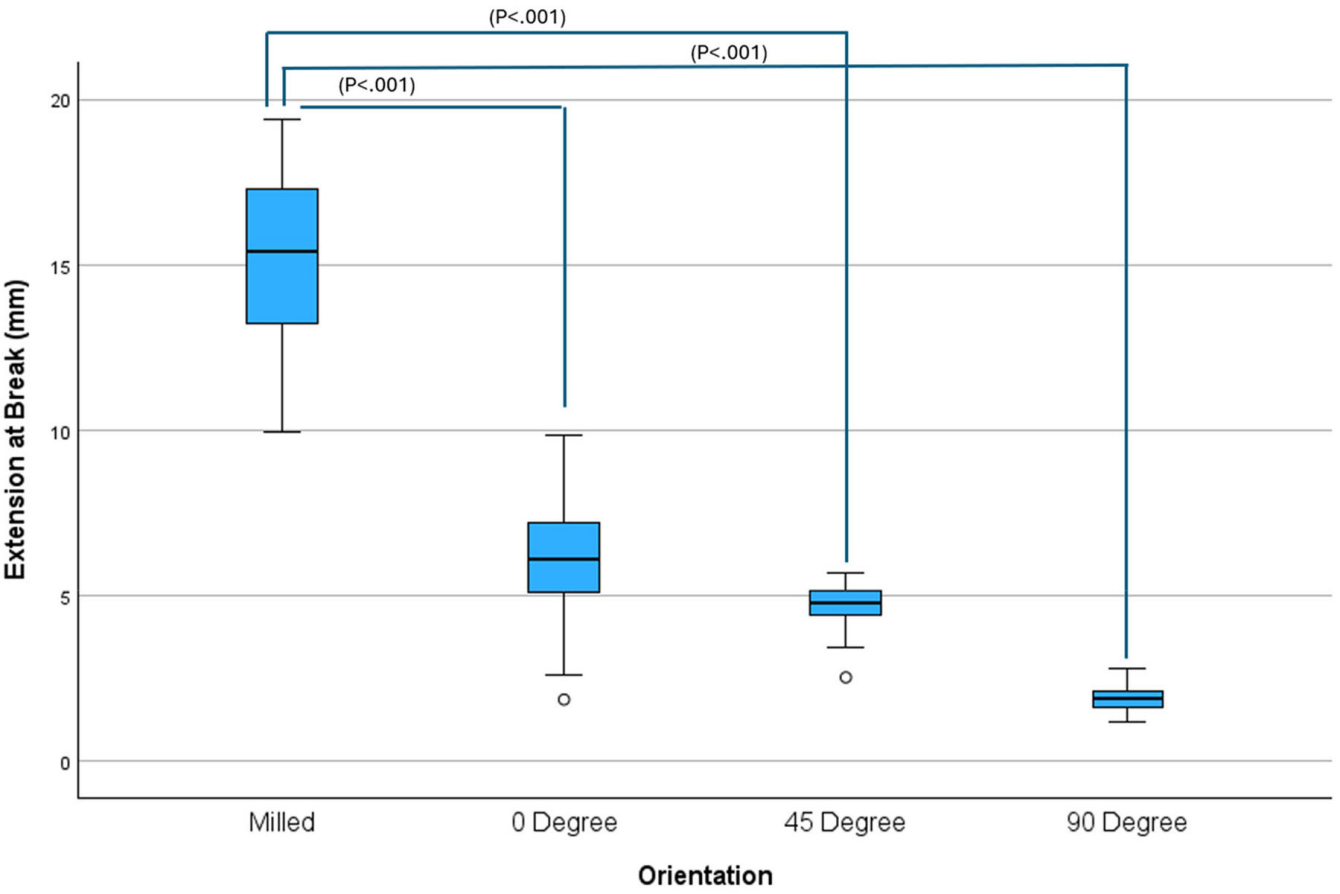
Extension at break.

Flexural strength is a key requirement for denture base materials, as they must endure repeated loading during mastication and resist fracture from accidental impacts (Alqutaibi et al. 2023). PMMA's well‐documented vulnerability to fracture under stress limits its performance as an ideal denture base material (Alqutaibi et al. 2023). The results of the present study suggest that PolyJet‐printed samples oriented at 0° can exceed the minimum ISO threshold for flexural strength and even outperform milled controls—supporting their potential as a viable alternative (Figure 5).

However, mechanical resilience cannot be assessed by flexural strength alone. Brittleness, indicated by extension at break, must also be considered. Denture fractures commonly result from accidental drops, and brittle materials are more prone to catastrophic failure under such conditions. In this study, extension at break was highest in the milled group (15.05 mm), nearly triple the value seen in the best‐performing printed group (5.99 mm at 0°). The 90° group demonstrated the lowest values for both flexural strength and extension at break, indicating poor performance across both metrics.

Together, these findings emphasize the importance of optimizing build orientation to improve both strength and flexibility in 3D‐printed denture base materials. While PolyJet technology shows considerable promise, additional refinement is needed to ensure long‐term clinical durability.

## 5 Conclusion

Print orientation significantly affected the mechanical properties of PolyJet‐printed denture base samples. The 90° orientation demonstrated significantly lower flexural strength and extension at break compared to the other groups. Notably, the 0° orientation outperformed the milled control in flexural strength but demonstrated greater brittleness, as evidenced by reduced extension at break.

These findings suggest that while PolyJet 3D printing holds promise for denture base fabrication, orientation‐specific optimization is crucial. Additional research is needed to better understand the implications of build orientation on long‐term clinical performance.

## Author Contributions

**Gregory Bennett:** conceptualization (lead), methodology (lead), formal analysis (lead), writing – review and editing (lead). **Alex Kohler:** writing – original draft (lead), investigation (lead), writing – review and editing (supporting).

## Conflicts of Interest

The authors declare no conflicts of interest.

## Data Availability

Data is available upon request from the corresponding author.
